# ICOnnecta’t: Development and Initial Results of a Stepped Psychosocial eHealth Ecosystem to Facilitate Risk Assessment and Prevention of Early Emotional Distress in Breast Cancer Survivors’ Journey

**DOI:** 10.3390/cancers14040974

**Published:** 2022-02-15

**Authors:** Joan C. Medina, Aida Flix-Valle, Ana Rodríguez-Ortega, Rosa Hernández-Ribas, María Lleras de Frutos, Cristian Ochoa-Arnedo

**Affiliations:** 1 E-Health ICOnnecta’t and Psycho-Oncology Services, Institut Català d’Oncologia, L’Hospitalet de Llobregat, 08908 Barcelona, Spain; jmedina1@uoc.edu (J.C.M.); aflixv@iconcologia.net (A.F.-V.); crodriguez@iconcologia.net (A.R.-O.); mrhernandez@bellvitgehospital.cat (R.H.-R.); mlleras@iconcologia.net (M.L.d.F.); 2 Department of Psychology and Education Sciences, Universitat Oberta de Catalunya, 08908 Barcelona, Spain; 3 Department of Clinical Psychology and Psychobiology, Universitat de Barcelona, 08035 Barcelona, Spain; 4 Psycho-Oncology and Digital Health, Health Services Research in Cancer, Institut d’Investigació Biomèdica de Bellvitge (IDIBELL), L’Hospitalet del Llobregat, 08908 Barcelona, Spain; 5 Department of Psychiatry, Hospital Universitari de Bellvitge, 08908 Barcelona, Spain; 6 Centro de Investigación Biomédica en Red Salud Mental (CIBERSAM), 28029 Madrid, Spain

**Keywords:** breast cancer, cancer survivors, internet-based intervention, patient monitoring, patient reported outcomes measures, psychosocial intervention, stepped-care

## Abstract

**Simple Summary:**

In current clinical practice, between one third and a half of patients diagnosed with cancer experience distress. Moreover, many of these psychosocial needs often remain unaddressed, although effective interventions exist. Nowadays, eHealth solutions like ICOnnecta’t offer new tools to overcome these limitations and improve access to treatment. This digital ecosystem has been proved to be feasible to implement, reaching good acceptability, use, and satisfaction between users. In addition, it allowed symptom monitoring in real time, facilitating preventive early interventions. Overall, fostering social support appears as a key to facilitate a resilient response after diagnosis.

**Abstract:**

Psychosocial interventions prevent emotional distress and facilitate adaptation in breast cancer (BC). However, conventional care presents accessibility barriers that eHealth has the potential to overcome. ICOnnecta’t is a stepped digital ecosystem designed to build wellbeing and reduce psychosocial risks during the cancer journey through a European-funded project. Women recently diagnosed with BC in a comprehensive cancer center were offered the ecosystem. ICOnnecta’t consists of four care levels, provided according to users’ distress: screening and monitoring, psychoeducation campus, peer-support community, and online-group psychotherapy. Descriptive analyses were conducted to assess the platform’s implementation, while multilevel linear models were used to study users’ psychosocial course after diagnosis. ICOnnecta’t showed acceptance, use and attrition rates of 57.62, 74.60, and 29.66%, respectively. Up to 76.19% of users reported being satisfied with the platform and 75.95% informed that it was easy to use. A total of 443 patients’ needs were detected and responsively managed, leading 94.33% of users to remain in the preventive steps. In general, strong social support led to a better psychosocial course. ICOnnecta’t has been successfully implemented. The results showed that it supported the development of a digital relation with healthcare services and opened new early support pathways.

## 1. Introduction

It is widely agreed that comprehensive oncological treatments should consider early educational and psychosocial preventive care [1] addressed to assess individualized psychosocial risk and reduce the impact of cancer on mental health [2,3]. However, only a minority of survivors are screened and referred to receive psychosocial support and treatment. Apart from the shortage of psycho-oncologists, other factors that have been highlighted are long wait lists; time or mobility restrictions; or poor early detection [1]. This situation is especially alarming given the availability of effective psychosocial interventions in cancer [4].

Several actions have been proposed to improve accessibility to educational and psychosocial care. One option is to restructure their contents and intensity, delivering services progressively depending on the individualized needs detected. In this sense, recent studies have introduced psychosocial stepped-care (SC) interventions in breast cancer (BC) [5]. Although these alternatives showed high acceptance (between 51.8–84%) and tend to be effective, comparing their results is complicated. Every SC program has a different number of levels, characteristics, professionals involved, and criteria to step up. In consequence, they all show variable results [6]. 

Another possibility is to improve access to psychosocial attention through Information and Communication Technologies (ICT), developing internet-based health (i.e., eHealth) interventions. Indeed, they have already shown their capacity to overcome many limitations expressed for conventional care [7]. In the last decade, several web and mobile platforms for screening, monitoring, or managing symptoms have been created, with BC being a common focus [8]. These tools have provided faster, easier, more intense, and convenient risk assessment means to identify warning signs [9]. Also, they have proved to improve self-management, to promote communication between patients and professionals, and to develop peer support [10]. These functionalities, inherent to eHealth, are facilitators of patients’ satisfaction and of improved acceptance and use rates [11]. 

According to the Unified Theory of Acceptance and Use of Technology, the acceptance of eHealth interventions could be described as participants’ intention to use the digital tool [12]. A recent systematic review showed that acceptance rates for mobile interventions in cancer range between 40–57% [10]. Regarding use, its definition and calculation accumulates less consensus. Even considering the several definitions of the concept, Cho and colleagues [10] concluded that 70–92% of patients use eHealth solutions at least once. It should be noted that interactive systems with professionals’ follow-up and direct communication between patient and practitioner are the ones with the highest use [10,13]. Another relevant indicator is attrition, understood as the proportion of patients opting out from treatment [14]. The eHealth research refers attrition rates between 13–60% depending on intervention characteristics [14,15,16]. 

Moving on to patient-reported outcomes, several meta-analyses and systematic reviews in cancer survivors have shown that internet-based interventions could improve emotional distress, quality of life (QoL), social support, and symptom self-management, among other clinical variables [11,17,18,19]. However, it is generally recognized that more solid evidence is needed. 

Certainly, there is a good deal of research in cancer reporting proper feasibility and clinical results for the independent use of SC interventions based on screening and monitoring, and online psychosocial treatments. However, only very few research groups have recently explored internet-based psychosocial SC interventions with special focus in prevention, showing heterogeneous results regarding psychological outcomes [20].

Building upon the evidence exposed, a European consortium has created a stepped eHealth ecosystem (https://oncommun.eu/; accessed on 14 January 2022), named ICOnnecta’t, that has the purpose of integrating screening and monitoring risk assessment tools with early stepped educational and psychosocial interventions for BC survivors during the acute phase of their illness (from diagnosis to the end of the primary treatments). ICOnnecta’t was developed because, when its first design started in 2017, we did not find any solution encompassing all these features and oriented to the specific population we were interested in targeting. The project is part of a Horizon 2020 proposal, under the name ONCOMMUN and funded by the European Institute of Innovation & Technology, which pursued to deploy new digital tools in cancer care, featuring a strong commitment to innovation. 

The present study aimed to examine the feasibility of ICOnnecta’t in a sample of target users during its first-year implementation. Secondary aims were to assess the psychosocial status of patients and measure their evolution in the first months within the program.

## 2. Materials and Methods

### 2.1. Study Design

This pilot study follows a quasi-experimental single-group longitudinal design. The inclusion of a control group was not considered at this stage, focused on feasibility. This article has been written following the SPIRIT statement [21].

All procedures performed in this study involving human participants were in accordance with the ethical standards of the institutional and research committee and with the 1964 Helsinki declaration and its later amendments. The protocol was approved by the Clinical Research Ethics Committee of the leading institution on the 25th of October 2018 (PR343/18).

### 2.2. Participants

Participants were recruited from a public healthcare institution specialized in cancer, located in north-eastern Spain. The first participant was recruited on the 15th of March 2019. Therefore, to analyze first-year results, the data tranche until the 14th of March 2020 was extracted. Eligibility criteria were: (1) adults (≥18 years), (2) diagnosed with a first episode of BC in the previous 3 months, (3) internet access and user-level skills, and (4) fluent in Catalan or Spanish. Patients were excluded and referred to more specialized care in the same hospital if they showed: (1) major depressive disorder, psychosis, or substance abuse; (2) autolytic ideation; or (3) impaired cognition.

### 2.3. Intervention

**Figure 1:**
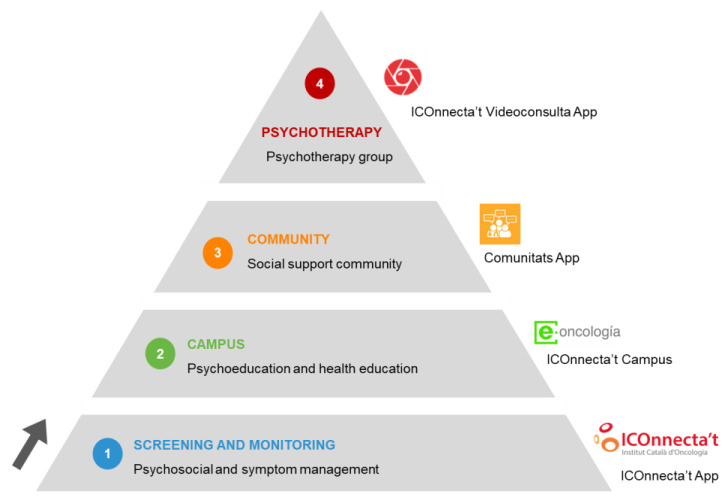
ICOnnecta’t stepped model.

ICOnnecta’t provides a SC intervention tailored to each patient throughout their cancer experience. It consists of 4 levels of care ordered by psychosocial complexity (see Figure 1). All patients enter the program at the first level and, whenever they step up, they retain access to the previous levels [22,23]. The details of the levels and the step-up protocol are outlined below: 

**-Level 1. Screening and monitoring symptoms and psychosocial risk assessment:** The first level is integrated in a central mobile application, named ICOnnecta’t, in which patients may connect and communicate with their health professionals about their psychosocial state and cancer’ symptoms, including treatments’ side effects. Thus, participants are monitored within ICOnnecta’t by health professionals with a traffic light system devised to this aim.

*Symptom management.* When participants report a symptom, a traffic light turns on. Its colors correspond to the symptom severity classification set by the National Cancer Institute in the guideline Common Terminology Criteria for Adverse Events (CTCAE) [24]. Green and yellow lights (CTCAE’s grade 1 and 2, respectively) mean low risk symptoms (e.g., hair loss), and orange and red lights (CTCAE’s grade 3 and 4, respectively) mean high risk (e.g., high fever). In all cases, participants receive tailored automatic health educational feedback from the system according to their symptom severity (i.e., health recommendations for symptom management), while for red lights it also shows the emergency services contact details. The health feedback messages were exhaustively developed by a working group composed of nurses, oncologists, nutritionists, and pharmacists from the healthcare institution. Apart from these automatic responses, the nurses of our team contact patients afterwards.

*Psychosocial care.* Patients are programmed psychometric questionnaires periodically in this platform to screen and follow-up their psychosocial evolution. To step up to the next intervention levels, the scores of an emotional thermometer (0–10 visual analogue scale (VAS)) are considered. This thermometer is administered weekly, and its use has been recommended in oncological settings to rapidly detect psychosocial morbidity [25], with a sensitivity of 70% and specificity of 73% in southern Europe for a ≥6 cut-off [26]. These scores are interpreted as moderate emotional distress (i.e., orange light), while those ≥8 as high (i.e., red light). If any of these warnings is flagged for at least 2 weeks, the Hospital Anxiety and Depression Scale (HADS) [27] is administered in addition to the routine schedule of this instrument. This 2 week delay is intentionally introduced to give the person time to explore the resources at each step. In case distress is confirmed (HADS ≥10) [28], a videoconference is scheduled to explore their needs and propose access to the second level of care (i.e., Campus). If the patient agrees, specific contents within the Campus are prescribed, tailoring the intervention to each of them. The same monitoring procedure is followed at all four care levels, and whenever distress is identified in the routine quarterly administrations of the HADS. Therefore, the patient journey in the ecosystem is always guided and accompanied by the healthcare team.

**-Level 2. Campus:** The second level of care offers a wide variety of educational resources through a virtual campus, across several topics that have been found relevant for cancer patients (e.g., lifestyle, mood, social relationships). All videos, posts, infographics, and articles have been selected and co-created with BC patients and health professionals. This level is available to patients in an unguided manner from the beginning to facilitate access to reliable information. Differently, when they step up to the second level of care, their Campus access is guided and therefore tailored to their needs.

**-Level 3. Communities:** The social community hosted in the third level of ICOnnecta’t, guided by health psychologists, is structured in 12 thematic areas mirroring the Campus co-created topics. Its main objective is to foster peer emotional and social support and to bridge the gap between users and their healthcare team, who stimulate debate and solve specific doubts. 

**-Level 4. Group psychotherapy:** The most intensive and specialized intervention in our digital ecosystem consists of group psychotherapy delivered through videoconference. It comprises eight weekly 90 min sessions, and is based on a positive psychology approach [29]. These sessions are led by a clinical psychologist specialized in psycho-oncology. 

ICOnnecta’t was developed through the cooperation between the hospital’s Information Technology Unit and technological providers, after the signature of a legal agreement to define data management and privacy standards compliant with the European General Data Protection Regulation (GDPR; EC/2016/679). Patients’ sensitive personal data remained hosted in the hospital’s secure servers in all cases, with communication means point-to-point encrypted to maintain confidentiality and datasets anonymized.

### 2.4. Acceptance, Use, and Attrition of the eHealth Intervention

*Acceptance.* Following reference guidelines [10,13], in the present study the acceptance rate of the digital platform was operationalized as percentage of enrolled patients, that is, number of participants who accepted the eHealth program divided by number of eligible patients to whom the program was proposed.

*Use*. The participants’ use rate was calculated based on the number of participants that reported their psychosocial status at least in one of the platform’s instruments divided by the total accepting participants [10].

*Attrition*. The attrition rate was expressed as the number of participants who informed on their willingness to stop using the platform after the initial acceptance divided by the total accepting participants. In other words, participants who opted out from the program [14].

### 2.5. Instruments

*Distress*. The HADS is a self-reported instrument to measure distress in individuals with a physical illness [27]. It is composed by two subscales, anxiety and depression. The Spanish validation in oncological patients proved high reliability (*α* = 0.82 for anxiety and *α* = 0.84 for depression). The HADS was administered routinely every three months and its overall score was considered the primary outcome, with scores ≥10 interpreted as moderate and ≥16 as high distress [28].

*Post-traumatic stress*. The Post-traumatic Stress Disorder Checklist for DSM-5 (PCL-5) is a self-report to measure post-traumatic symptoms [30]. It includes 20 items and has shown good reliability (*α* = 0.94). The official Spanish version has not been published yet, but it was provided by the United States National Center for Post-Traumatic Stress Disorder for the purpose of this study. The PCL-5 was administered every three months, with scores ≥33 interpreted as moderate and ≥45 as high stress [31].

*Post-traumatic growth*. The Post-traumatic Growth Inventory (PTGI) is a self-reported measure for growth experiences following traumatic events [32]. It features 21 items and has been validated in Spain with an oncological sample, proving high reliability (*α* = 0.95). The PTGI was administered every three months, with scores ≥46 interpreted as high growth [33].

*Quality of life*. The EuroQoL-5D-3L (EQ-5D-3L) is a brief instrument to measure QoL [34]. It covers five dimensions related to both physical and mental health. The Spanish version has shown appropriate convergent and construct validity, and was administered every three months in our sample with time trade-off scores ≥90 interpreted as high QoL [35]. 

*Social support*. Participants’ perceived social support was measured with the Medical Outcomes Study—Social Support Survey (MOS-SSS) [36]. The Spanish version has been validated with cancer patients and it showed excellent reliability (*α* = 0.94). No cut-off scores are applicable to this instrument.

*Satisfaction and usability*. Satisfaction with the digital ecosystem and its perceived usability were assessed three weeks after registration with a 0–10 VAS. No clear cut-offs have been found in the literature, we interpreted their scores ≥5 as some and ≥8 as high satisfaction/usability.

Sociodemographic and clinical data were collected from patients’ clinical records after obtaining their informed consent.

### 2.6. Procedure

All new patients treated in the BC unit of the recruiting institution were informed of the study by their nurses. Those showing interest met with an ICOnnecta’t team member to discuss about the program, check eligibility criteria, and sign the informed consent. Then, participants were guided to install the digital ecosystem on their devices and were offered a basic training to use it. Thereafter, the screening and monitoring of the participants (i.e., level 1) could start. Participants who accepted the enrollment but finally did not make use of the eHealth platform received usual face-to-face care in the health institution (i.e., medical and nursing follow-up visits, referral to psychological care only if distress is inferred by health professionals). 

### 2.7. Statistical Analyses

The *R* software was used [37]. First, descriptive analyses were conducted to appraise the implementation of the digital ecosystem, covering its acceptance, use and attrition rates, as well as user satisfaction and usability. Differences between participants who used the system and those who did not were also estimated with Chi-squared and Student’s *t* tests as appropriate. Then, the platform’s functioning was assessed according to the number of health education and psychosocial needs detected, and the time needed to provide care, in addition to users’ distribution across its SC program.

Finally, we were interested in understanding patients’ psychosocial course immediately after diagnosis. Therefore, we conducted independent multilevel linear models (MLM) for all outcomes of interest (i.e., HADS, PCL-5, PTGI, and EQ-5D-3L). For each model, we included only those participants who provided at least one score during the first month immediately after inclusion and analyzed their evolution in the 3 months afterwards.

Models were built parsimoniously and considered maximum likelihood as estimation method. We always started by the simplest meaningful model with fixed intercept and time, and increased complexity progressively in nested models supported by likelihood ratio tests (LRT). In these subsequent models, we added social support at baseline and sociodemographic variables (i.e., age, marital status, education, and work status) as predictors. We were especially interested in social support since its role in psychological adjustment after diagnosis has been repeatedly highlighted [38,39]. Results reported herein are those for the best-fitting model for each outcome. The covariance structures that best fitted our data were first-order autoregressive.

## 3. Results

### 3.1. Participant Characteristics 

**Figure 2:**
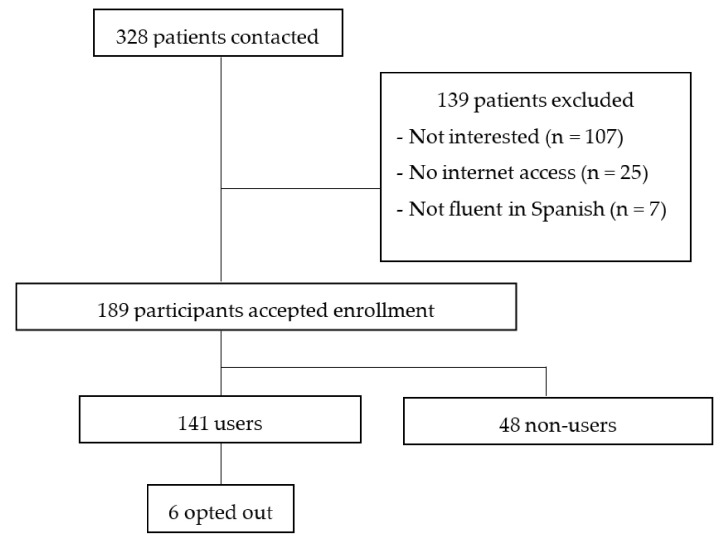
Participants’ flowchart.

Up to 328 patients were referred to ICOnnecta’t from the BC unit of the recruiting institution in the time frame of the study (i.e., one year). Among these, 189 patients were finally enrolled, which entails an acceptance rate of 57.62%. Moreover, 141 participants completed at least one of the scheduled instruments, which represents a use rate of 74.60%. The other 48 participants (25.40%) did not make use of the platform and no outcome data could be extracted from them. In turn, the attrition among users was of 4.26% (*n* = 6). All six participants lost interest in the ecosystem. Added together non-users and those users who opted out, global attrition can be established at 29.66%. The participants’ flowchart can be seen in Figure 2.

Finally, the average satisfaction level with the platform among users was 6.22 (SD = 3.17), although only 63 participants answered this instrument. Up to 76.19% (*n* = 48) reported being satisfied, with 41.27% (*n* = 26) specifically very satisfied. The mean platform usability perceived by users was 7.09 (SD = 3.77), estimated with 79 respondents. For 75.95% (*n* = 60), the ecosystem was easy to use. Particularly, most of them (68.35%, *n* = 54) reported it as very usable.

Participants’ main sociodemographic and clinical characteristics are shown in Table 1. No significant differences existed between users and non-users. However, it can be noticed that the former were slightly younger and more educated. 

### 3.2. Symptom and Psychosocial Management

During this first year, 150 symptoms requiring attention (i.e., orange and red lights) were detected from 61 participants. The average time elapsed from patients’ report and professionals’ first response was 2.05 days (SD = 4.14). In turn, up to 293 psychosocial warnings (i.e., orange and red lights) were identified from 71 individuals, with an average of 5.91 days (SD = 7.83) from patients’ report and initial support provided by psychologists in the team.

Most of these needs could be solved after this initial care, with only 43 symptoms and 48 psychosocial needs requiring further attention. These contacts were conducted on average 0.12 days (SD = 0.6) after the first response in case of symptoms, and 7.15 days (SD = 11.1) for psychosocial warnings. This last estimation was extended by the two-week interval set prior to decide whether to refer patients to higher levels of care.

Indeed, as for the escalation rates of the digital ecosystem, up to 102 (72.34%) of the 141 participants did not require further intervention beyond the first level of ICOnnecta’t. For the remaining 39 individuals (27.66%), persistent distress was detected and were given access to the second level of care (Campus). Then, 15 (10.64%) continued experiencing distress at this level and were referred to the third (Communities) step. Finally, eight (5.67%) reached the fourth and most intensive level (group psychotherapy).

### 3.3. Users’ Psychosocial Course in ICOnnecta’t after Diagnosis 

Up to 14.74% of participants were found high and 31.58% moderate distress in the HADS, whereas 13.33% scored high and 10.67% moderate stress in the PCL-5. On the contrary, 43.48% of participants already showed high growth in the PTGI, despite 57.28% scored below the QoL cut-off score in the EQ-5D-3L. Finally, participants reported strong social support in the MOS-SSS, with an average exceeding by ten points the mean estimations reported in the literature [40,41]. Mean scores obtained by participants in all instruments can be seen in Table 2.

The MLM results for the HADS (*n* = 95) showed significant variance in its intercepts across participants (*χ*^2^(1) = 24.85, *p* < 0.001), and included time and social support as fixed predictors. Time did not yield a significant effect (*b* = −0.012, *p* = 0.077, 95% CI = −0.025 to 0.001), but social support did (*b* = −0.251, *p* < 0.001, 95% CI = −0.334 to −0.167), with lower scores in the HADS associated with higher support. 

For the PCL-5, the MLM (*n* = 75) showed again significant variance in participants’ intercepts (*χ*^2^(1) = 52.54, *p* < 0.001), and included time and social support as fixed. Time was not significant (*b* = 0.017, *p* = 0.462, 95% CI = −0.028 to 0.061), but social support was (*b* = −0.539, *p* < 0.001, 95% CI = −0.813 to −0.265), as high scores predicted lower levels in the PCL-5.

Moving on to the PTGI model (*n* = 46), intercepts varied across participants (*χ*^2^(1) = 8.59, *p* = 0.003) and included time and social support as fixed predictors. Like in the previous models, time did not prove to be significant (*b* = 0.083, *p* = 0.265, 95% CI = −0.068 to 0.234). For the PTGI, social support was not either (*b* = -0.146, *p* = 0.704, 95% CI = −0.896 to 0.604).

Finally, in the EQ-5D-3L model (*n* = 103), intercepts did not show significant variance *χ*^2^(1) = 1.46, *p* = 0.228), so it included fixed intercept and time, which again, was not a significant predictor of EQ-5D-3L scores (*b* = −0.001, *p* = 0.127, 95% CI = −0.001 to 0.001).

## 4. Discussion

This study sought to report, for the first time, the implementation and initial results of ICOnnecta’t, an eHealth ecosystem designed to deliver preventive education and psychosocial care in cancer based in individualized risk assessment. The acceptance and use rates were within the expected ranges, with around half of the patients accepting the platform and, among these, three quarters actually using it [10]. In our sample, there was a tendency in users to be slightly younger and to have higher studies than non-users, but the non-significance of these results strengthen the idea that sociodemographic barriers for eHealth use are vanishing [42]. 

In turn, attrition was relatively low and within the expected ranges, which is a strength of the program, but needs to be tested with more diagnoses, since attrition in BC tends to be particularly low [15]. In addition, most users reported high levels of both satisfaction with, and usability of, the platform. Although not all of them provided their views on these matters, ICOnnecta’t seems to tackle some of the main limitations found among cancer survivors’ experience with telehealth through personalization, trusting relationships, and patient autonomy favored by symptom self-management [11]. Indeed, other recent eHealth interventions have obtained similar results in terms of satisfaction and ease of use [9].

Regarding the detection of needs and the provision of appropriate care, the digital ecosystem proved to facilitate patients’ follow-up as it has already been reported for eHealth [8] and SC interventions [6]. An average of two days for health education, and under six for psychosocial warnings, were established as waiting-times to be provided with a professional first response. In addition, this fast management was also efficient since most needs could be solved with a quick initial reply. This finding needs to be merged with the fact that 94.33% of participants remained within the (preventive) first three levels of the SC program. Therefore, ICOnnecta’t may be a valuable complement, and even an alternative, to usual care. It is relevant to highlight in this regard that this digital ecosystem does not seek to replace healthcare professionals, but to provide them with more responsive means to deliver care. Indeed, the program pursues to foster the collaboration within the patient-professional dyad, which articulates healthcare decisions in all cases.

The prevalence of distress found among patients after diagnosis was aligned with the literature [38] with moderate rather than high scores. Similarly, the low proportion of stress was also coincident with extant research for BC [43], and the same applied for the moderate levels of posttraumatic growth [44]. Regarding QoL, results proved most patients to score below the population cut-off point at baseline [35], findings that must be interpreted considering that participants were starting their primary oncological treatments. This condition impacts on several areas covered by the EQ-5D-3L, such as the feeling of pain and the performance of daily routines [45]. 

Finally, strong social support was perceived by most participants. This finding is of interest given its stress-buffering role [39,45]. Indeed, in our longitudinal analyses we consistently found stronger social support to be associated with better results, underlining the relevance of this variable. Fostering fulfilling and supportive relationships seems to be a key factor for attaining a better course and, if assessed from the beginning, it may anticipate psychosocial trajectories during treatments. This finding confirms that the peer support community featured in ICOnnecta’t could be one of its main assets. 

It is true that, unlike other similar studies [6,20], we have not found significant improvements with time. However, these previous proposals often included only participants who were already experiencing distress, while we offered the platform to all new patients with a health promotion objective. The fact that the majority of users showed a resilient response to cancer makes significant improvements unlikely to occur, as they are typically found among patients with a poorer mental health status at baseline [6]. Consequently, in our sample a steady trend means most users remain free from clinical symptoms, even when active treatments are still in play [39,45].

Apart from the small sample size and the short-term longitudinal data collected, the present work has other limitations. Although this was a feasibility study, the absence of a control group limits the interpretation of results. In addition, not all patients completed all questionnaires, as they were reminded of the importance of doing so, but did not receive any kind of pressure given the real-world nature of the project. Also, we could not extract any data from the non-users who accepted to participate, but finally did not make use of the platform, who could not be provided care through it either. These patients continued to receive usual in-person care in the hospital, they attended their medical and nursing periodic visits, but did not receive any psychosocial support unless the healthcare team perceived distress during their appointments. However, since we could not measure any outcome variable from them, no comparisons between the effect of ICOnnecta’t and usual care could be made. The results of future randomized controlled studies are expected to contribute to this line of research. Similarly, although both patients and practitioners were involved in the design of the eHealth solution, we focused on the former at this stage and did not administer any instrument to the professionals involved in its implementation. In the future, it may be relevant to study their insights as well, both in terms of patients’ progress and regarding their own satisfaction with, and usability assigned to the system. Finally, although we found no sociodemographic differences between users and non-users, we must acknowledge that the 25 patients reported in Figure 2 to reject participation due to no internet access might have changed these results. However, since they represented only 7.62% of all individuals who were offered the ecosystem, such influence is still mild.

## 5. Conclusions

In conclusion, this study supports the use of eHealth in BC healthcare, with preliminary results suggesting the absence of sociodemographic barriers for their acceptance and use. ICOnnecta’t allowed professionals to timely monitor and manage needs throughout patients’ journey, intervening before the clinical course of physical and psychological symptoms worsen. This feature has the capacity to prevent suffering in patients, also saving costs for health systems. While many users show a resilient response to their diagnosis, many others do not. Consequently, it will be clinically relevant to refine the individualized risk assessment to identify and model differential trajectories among the whole sample in the future, in order to feed more precise and personalized treatments and to better estimate effectiveness and cost-utility [22]. Future studies will also aim to replicate these results and to extend ICOnnecta’t to other diagnoses and countries, making its services available to more patients who may benefit from them. Indeed, preliminary versions of this digital ecosystem have already been developed in Portuguese and Polish, following a careful adaptation process to each cultural background. We hope this work will lead to increase the availability of comprehensive cancer care programs in more regions.

## Figures and Tables

**Table 1:** Demographic and clinical characteristics of users and non-users.

	Users (*n* = 141)	Non-Users (*n* = 48)	*t*	*X* ^2^	*p*
Age M (SD)	52.35 (8.57)	55.15 (9.55)	1.90		0.059
Marital status *n* (%)				0.87	0.929
Single	9 (6.38)	2 (4.17)			
Married/partnered	101 (71.63)	33 (68.75)			
Divorced/separated	6 (4.26)	3 (6.25)			
Widowed	2 (1.42)	1 (2.08)			
Unknown	23 (16.31)	9 (18.75)			
Education *n* (%)				7.30	0.063
Primary or no studies	5 (3.55)	2 (4.17)			
Secondary	17 (12.06)	3 (6.25)			
Tertiary	43 (30.50)	7 (14.58)			
Unknown	76 (53.90)	36 (75.00)			
Work status *n* (%)				5.83	0.323
Active	54 (38.30)	12 (25.00)			
Passive	13 (9.22)	6 (12.50)			
Occupational disability	4 (2.84)	3 (6.25)			
Work leave	21 (14.89)	5 (10.42)			
Retired	9 (6.38)	6 (12.50)			
Unknown	40 (28.37)	16 (33.33)			
Cancer stage *n* (%)				3.77	0.438
0	16 (11.35)	3 (6.25)			
I	53 (37.59)	22 (45.83)			
II	52 (36.88)	15 (31.25)			
III	15 (10.64)	4 (8.33)			
IV	5 (3.55)	4 (8.33)			

**Table 2:** Participants’ mean scores and standard deviations after diagnosis.

	Mean	SD
HADS	9.89	6.52
PCL-5	24.6	15.6
PTGI	37.8	23.9
EQ-5D-3L	0.82	0.22
MOS-SSS	81.4	12.1

HADS: Hospital Anxiety and Depression Scale; PCL-5: Post-traumatic Stress Disorder Checklist for DSM-5; PTGI: Post-traumatic Growth Inventory; EQ-5D-3L: EuroQoL-5D-3L; MOS-SSS: Medical Outcomes Study—Social Support Survey.

## Data Availability

The anonymized datasets of this study may be obtained from the corresponding author upon reasonable request.
